# Evaluating the impact of GLP-1 receptor agonists in combination with total neoadjuvant therapy for locally advanced rectal cancer

**DOI:** 10.1093/bjs/znag029

**Published:** 2026-03-10

**Authors:** Hugo C Temperley, Matthew Coalter, Ben Creavin, Patrick Jordan, Andrew Hogan, Jacintha O’Sullivan, Donal O’Shea, Paul H McCormick, Emily Harrold, Michael E Kelly

**Affiliations:** Department of Radiology, St. James’s Hospital, Dublin, Ireland; Trinity St. James’s Cancer Institute, St. James’s Hospital, Dublin 8, Ireland; Department of Surgery, St. James’s Hospital, Dublin 8, Ireland; Department of Immunology, St. James’s Hospital, Dublin, Ireland; Department of Surgery, St. James’s Hospital, Dublin 8, Ireland; Department of Surgery, Royal Brisbane and Women’s Hospital, Brisbane, Queensland, Australia; Department of Surgery, St. James’s Hospital, Dublin 8, Ireland; Obesity Immunology Research Group, Maynooth University, Maynooth, Ireland; Trinity St. James’s Cancer Institute, St. James’s Hospital, Dublin 8, Ireland; School of Medicine, Trinity College Dublin, Dublin 2, Ireland; Department of Endocrinology, St Vincent's University Hospital, Dublin 4, Ireland; Department of Surgery, St. James’s Hospital, Dublin 8, Ireland; Department of Medical Oncology, St. James’s Hospital, Dublin 8, Ireland; Trinity St. James’s Cancer Institute, St. James’s Hospital, Dublin 8, Ireland; Department of Surgery, St. James’s Hospital, Dublin 8, Ireland; School of Medicine, Trinity College Dublin, Dublin 2, Ireland

## Abstract

Total neoadjuvant therapy (TNT) has become a standard treatment approach for rectal cancer, providing higher rates of pathological complete response and improved long-term survival. Glucagon-like peptide-1 receptor agonists (GLP-1 RAs) have shown significant advantages in weight loss, systemic metabolic regulation, and anti-inflammatory effects. Emerging evidence also points to possible anticancer properties, with observational data suggesting a lower incidence of obesity-related cancers, including colorectal cancer. This narrative review aims to examine the biological basis and potential therapeutic benefits of combining GLP-1 RAs with TNT for the management of locally advanced rectal cancer. We explore how GLP-1 RAs may affect tumour biology and treatment tolerance, including their impact on visceral fat, insulin resistance, and systemic inflammation. Preclinical and clinical data are reviewed to determine whether GLP-1-induced metabolic changes can improve the effectiveness of chemotherapy and enhance surgical and oncological results. Although evidence is evolving, the integration of GLP-1 receptor agonists into rectal cancer treatment pathways represents a promising area for further investigation, particularly in metabolically vulnerable populations.

## Teaser

Total neoadjuvant therapy (TNT) is now standard for locally advanced rectal cancer, yet metabolic factors may influence treatment response and tolerance. This narrative review explores the biological rationale and emerging evidence for combining glucagon-like peptide-1 receptor agonists (GLP-1 RAs) with TNT, focusing on their effects on tumour biology, systemic inflammation, insulin resistance, and visceral adiposity. Although clinical data remain limited, GLP-1 RAs represent a promising adjunctive strategy to enhance multimodal rectal cancer care, particularly in metabolically vulnerable patients.

## Introduction

Rectal cancer comprises approximately 30–40% of colorectal adenocarcinoma, the third most common solid organ malignancy worldwide^1^. One-third of this is locally advanced rectal cancer (LARC)^2^, defined as disease that has invaded through the muscularis propria up to but not beyond the rectal serosa (T3), or through the wall of the rectum into adjacent organs (T4), and/or involving mesorectal lymph nodes in the absence of metastatic disease^3^. The current guidelines in the UK and Ireland^4^, Europe^5^, and the United States^3^ are to treat locally advanced rectal cancer with total neoadjuvant chemoradiotherapy (TNT) followed by total mesorectal excision (TME)^6^. TNT has been shown to improve overall survival (OS) and disease-free survival (DFS), increase the pathological complete response rate (pCR), reduce toxicity, improve treatment adherence, and increase sphincter preservation^7–10^.

Obesity is a recognized negative prognostic factor in colorectal cancer, with affected patients demonstrating poorer clinical outcomes^11^. Patients with obesity have higher rates of all-cause morbidity (43% *versus* 21%)^12^, as well as higher rates of recurrence^13^. Weight loss prior to surgery has been proposed to mitigate these risks by improving patients’ functional status. Traditionally, a structured approach to weight loss with a prehabilitation programme of exercise, nutritional supplementation, and counselling has demonstrated the greatest benefit, but this is very challenging for patients who are also undergoing intensive neoadjuvant treatment, and compliance can be an issue^14,15^.

A growing proportion of the U.S. population is using glucagon-like peptide-1 receptor agonists (GLP-1 RAs) for type 2 diabetes and weight loss. The influence of these drugs on whole body and cancer cell metabolism is significant^16^. GLP-1 RAs, such as semaglutide, have shown potent effects on weight loss in individuals living with obesity. In the STEP 1 trial, semaglutide induced an average weight loss of 14.9% compared to 2.4% with placebo over 68 weeks^17^. Recent epidemiologic data also suggest a link between GLP-1 RA use and reduced incidence of obesity-related cancers, including colorectal cancer^18^. Additionally, GLP-1 RAs may have anti-inflammatory and metabolic effects that could benefit cancer therapy. The aim of this review is to examine the existing literature and summarize the current evidence for the potential impact of GLP-1 RAs in TNT for locally advanced rectal cancer.

## Methodology

This narrative review was conducted using a structured literature search of PubMed/MEDLINE, Embase, and Web of Science databases, encompassing publications from January 2000 to January 2025. Search strategies incorporated combinations of the following keywords and Medical Subject Headings (MeSH) terms: ‘GLP-1 receptor agonist’, ‘semaglutide’, ‘obesity’, ‘metabolism’, ‘rectal cancer’, ‘colorectal cancer’, ‘neoadjuvant therapy’, and ‘total neoadjuvant therapy’. Eligible publications included preclinical investigations, observational cohort studies, RCTs, and relevant systematic reviews published in English.

## Total neoadjuvant therapy in rectal cancer

Multiple RCTs ave provided high-level evidence that TNT improves clinical outcomes in LARC compared with conventional treatment approaches. The Preoperative Chemotherapy for Patients with Locally Advanced Rectal Cancer (PRODIGE-23) trial showed improved DFS and lower distant metastases at 3 years, and is the only study to demonstrate an OS advantage^10,19^. A recent systematic review and network meta-analysis of all the published TNT protocols to date demonstrated that long-course chemoradiotherapy followed by consolidation chemotherapy had the highest pCR as well as the most favourable toxicity profile when compared to the other regimens^8^.

TNT encompasses heterogeneous, evidence-based regimens that differ by radiotherapy modality, chemotherapy backbone, and sequencing strategy^19–21^. Given that these variables influence treatment duration, gastrointestinal toxicity, and systemic catabolic stress, they may plausibly modify metabolic and inflammatory responses. Therefore, any trial evaluating GLP-1 RAs alongside TNT should predefine the TNT platform and stratify (or adjust) for radiotherapy type, sequencing (induction *versus* consolidation), and type of chemotherapy, to ensure biological interpretability and avoid confounding by regimen heterogeneity.

Any consideration of GLP-1 receptor agonist integration must be situated within the rapidly evolving therapeutic landscape of locally advanced rectal cancer. Contemporary management increasingly emphasizes organ preservation strategies, including total neoadjuvant therapy-based non-operative management (‘watch-and-wait’) in patients achieving a clinical complete response^22,23^. In parallel, immunotherapy has transformed the treatment paradigm for patients with deficient mismatch repair / microsatellite instability–high (dMMR/MSI-H) rectal cancer, with PD-1 blockade demonstrating unprecedented complete response rates that may obviate the need for chemoradiotherapy or surgery in selected populations^24^. Within this context, GLP-1 receptor agonists should not be conceptualized as tumour-directed agents, but rather as metabolic adjuncts that may enhance treatment tolerance, optimize body composition, and potentially modulate systemic inflammatory milieu during cytotoxic therapy. Importantly, their proposed role would be limited to mismatch repair proficient disease undergoing TNT, and would not interfere with established immunotherapy pathways in dMMR/MSI-H cohorts. Future studies should therefore carefully define molecular subtype and treatment paradigm when evaluating metabolic adjunct strategies in rectal cancer.

## GLP-1 RAs: mechanisms and clinical applications

### What are GLP-1 RAs?

GLP-1 RAs are synthetic versions of the GLP-1 hormone^25^. The natural hormone GLP-1 binds to the GLP-1 receptor and helps regulate blood sugar levels and satiety signals. This function makes it an ideal target for treating type 2 diabetes (T2D) and obesity. GLP-1 RAs have had a transformative impact on the management of T2D. The GLP-1 receptor is primarily expressed on pancreatic β cells. In the presence of glucose, this binding stimulates insulin release and suppresses glucagon secretion, which improves glucose uptake in the periphery^26^. In non-human primates, GLP-1 receptor expression was detected in brain regions involved in regulating food intake, such as the hypothalamus and brainstem^27^, helping to explain the effects of GLP-1 RAs in reducing addictive behaviours^28^. GLP-1 RAs have been shown to slow gastric emptying, an effect believed to result from post-prandial inhibition of myenteric neurons of the gastrointestinal tract, involving nitric and cyclic adenosine monophosphate-dependent mechanisms^29^. The delay in gastric emptying contributes to both a reduction in food intake and side effects like nausea, which is common and leads to discontinuation of therapy in 10–20% of patients^30^. Due to these combined effects, most patients with T2D using GLP-1 RAs experience improved fasting serum insulin and insulin sensitivity, lower haemoglobin A1c (HbA1c) levels, and reduced reliance on supplemental insulin^31,32^. Recent polls suggest that 6% of adults in the United States use GLP-1 RAs, despite high costs and drug shortages^33^. Their significant benefit is dependent on the potency of the agent used and the trials, but data show they can cause weight loss of between 5% and 18% of body weight in obesity^34^ and a reduction in HbA1c of up to 2.1%^31^. The safety profile of GLP-1 RAs is generally favourable; most adverse events are gastrointestinal (nausea, diarrhoea, vomiting), which can be managed with dose titration.

### GLP-1 RA’s impact on cancer risk

Mavromatis *et al*. studied 85 000 patients and found a 16% lower colon cancer and a 28% lower rectal cancer rate in GLP-1 RA users compared with dipeptidyl peptidase-4 (DPP-4) inhibitors^35^. Although these are observational findings, their consistency across large datasets strengthens the hypothesis that GLP-1 RAs have protective effects against colorectal cancer (CRC). Beyond CRC, GLP-1 RAs seem to protect against various obesity-related cancers. The group mentioned previously also found a 7% decrease in overall obesity-associated cancers and an 8% lower mortality rate among female patients on GLP-1 RAs^35^. Wang *et al*. reported a 19% reduction in 13 obesity-related cancers, with half the 15-year mortality rate in those treated with GLP-1 RAs^18^. Whether this is solely related to their benefits in weight loss and improved glycaemic control or whether they have an additional antitumour effect will be explored.

### T2D and obesity in cancer

Hyperglycaemia and obesity are both chronic risk factors for cancer development and progression^36^. Hyperglycaemia leads to the formation of advanced glycation end-products (AGEs), which bind their receptors and cause proinflammatory signalling and cellular stress^37^. Hyperglycaemia and insulin resistance cause high circulating insulin-like growth factor 1 (IGF1) and insulin levels, both of which are pro-oncogenic through pathways such as phosphoinositide 3-kinase–protein kinase B–mechanistic target of rapamycin pathway (PI3K-AKT-mTOR) and RAS–mitogen-activated protein kinase pathway (RAS-MAPK). These promote new blood vessel growth (that is they are angiogenic) leading to enhanced tumour growth^38^. Angiogenesis is essential to supply tumours with their nutrients and oxygen to grow and enlarge and allows them to penetrate blood vessels to metastasize. Hyperglycaemia has also been linked to immune evasion in the tumour microenvironment (TME) in several ways. One interesting pathway is through glucose-induced *O*-GlcNAcylation in tumour-associated macrophages (TAMs), pushing them towards an M2 phenotype and away from cancer cell destruction^39^. These cells inhibit adaptive antitumour immunity through mediators like interleukin 10 (IL-10) and transforming growth factor beta (TGFβ)^40^. A small study of specimens from diabetic patients with colorectal cancer had increased numbers of M2-like macrophages compared to colorectal cancer patients without diabetes, further suggesting that hyperglycaemia can promote this switch away from antitumour immunity. These harmful effects of uncontrolled hyperglycaemia support an argument for improved glycaemic control in patients with diabetes and cancer^39^.

Obesity can promote chronic inflammation through various pathways. Excess adipocytes release pro-inflammatory cytokines (such as tumour necrosis factor alpha (TNF-α), interleukin 6 (IL-6), and interleukin 1 beta (IL-1β)), fostering low-grade inflammation. Higher levels of circulating free fatty acids and glucose lead to high activity of oxidative pathways, over-activating mitochondria and releasing reactive oxidative species. These, combined along with others, can over-activate the immune system through signalling pathways like Nuclear factor kappa B (NF-κB) and c-Jun N-terminal kinase (JNK), causing both tumour initiation and progression^41,42^. In obesity, this TME pushes macrophages towards their immunosuppressed state and promotes tumour progression and metastasis^43^.

## Rationale for combining GLP-1 RAs with TNT

### GLP-1 RAs as an adjunctive therapy in cancer

GLP’s antitumour activity may go beyond obesity and weight loss. *In vitro* and limited *in vivo* data point towards stimulation of the GLP-1 receptor, inhibiting tumour growth and metastasis directly. In human pancreatic cancer cell lines, liraglutide attenuated growth and promoted apoptosis, and had a similar effect in a mouse xenograft model. This increased production of cyclic adenosine monophosphate (cAMP) results in the inhibition of Akt and ERK1/2 signalling pathways^44^, which are key for cancer cell survival, growth, metabolism, migration, and angiogenesis. Inhibition of Akt was in a liraglutide dose-dependent manner^45^. In mouse colon cancer cells that expressed GLP-1, exenatide increased cAMP and reduced signalling kinases glycogen synthase kinase 3 and ERK1/2 in CT26 cells, causing apoptosis and inhibiting proliferation^46^. In another preclinical study, liraglutide was found to directly inhibit colorectal cancer cell proliferation, migration, and invasion, while promoting apoptosis via inhibition of the PI3K/AKT/mTOR signalling pathway. This resulted in cell cycle arrest and reduced tumorigenic behaviours^16^.

There is evidence to support a synergistic effect between GLP-1 RAs and chemotherapy in anticancer regimes. In colon cancer cell lines, exenatide augmented apoptosis induced by the chemotherapeutic agent irinotecan when given together^46^. In another experiment, although gemcitabine-resistant pancreatic tumour cells expressed less GLP-1 receptor compared with non-resistant cells, treatment with liraglutide increased GLP-1 RA expression and inhibited growth and promoted apoptosis. This effect was neutralized by blocking GLP-1 receptor or NF-κB signalling. This paper also found liraglutide to increase the chemosensitivity of PANC-1 pancreatic tumour cells to gemcitabine, reducing cell growth and inducing more apoptosis when given together compared to gemcitabine treatment alone. In a mouse model, co-delivery of these drugs led to smaller tumour volume and weight than giving chemotherapy alone^47^. In a study of 33 cancer cell lines, GLP-1 signalling was found to be altered in most, and lower signalling associated with lower immune cell infiltration, higher tumour mutation burden, microsatellite instability, lower immunotherapy response and ultimately lower survival. The same paper found that colon cancer cell lines treated with semaglutide showed reduced migration, and increased activation of a gene called *ITPR1*, which was previously linked to paclitaxel cytotoxicity^48^, again suggesting a synergistic effect of co-therapy. The metabolic, hormonal, immunologic, and receptor-specific mechanisms likely work together, providing a biological basis for the broad anticancer associations seen across epidemiologic studies (Table 1).

**Table 1:** Biological, epidemiological, and translational rationale for integrating GLP-1 receptor agonists into TNT protocols for rectal cancer

Domain	Key evidence	Implications for TNT and rectal cancer
Metabolic effects	STEP-1 trial: semaglutide achieved 15% mean weight loss *versus* 2.4% with placebo; HbA1c reductions up to 2.1%	Weight reduction improves surgical feasibility and lowers perioperative morbidity rate; glycaemic control reduces tumour-promoting hyperinsulinaemia and inflammation
Cancer epidemiology	GLP-1 RA use linked to 16% ↓ colon cancer and 28% ↓ rectal cancer risk *versus* DPP-4 inhibitors. Wang *et al*.: 19% ↓ in obesity-related cancers, halved 15-year mortality rate	Large population data sets support an anticancer signal; rationale for exploring GLP-1 RAs in rectal cancer therapy
Preclinical antitumour activity	*In vitro*: GLP-1 RAs inhibit proliferation, induce apoptosis (↑ cAMP, ↓ AKT/ERK). *In vivo*: reduced tumour growth, enhanced dendritic cell activity, ↑ CD8+ T cells, ↓ Tregs	Suggests tumour-suppressive and immune-modulating effects; supports potential synergy with cytotoxic TNT
Chemotherapy synergy	Exenatide augmented irinotecan-induced apoptosis in colon cancer cells. Liraglutide enhanced gemcitabine sensitivity in pancreatic models. Semaglutide activated *ITPR1*, linked to paclitaxel cytotoxicity	GLP-1 RAs may potentiate TNT chemotherapy, improving pathological complete response rates and tumour regression
ctDNA as biomarker	Meta-analysis: ctDNA negativity after neoadjuvant therapy → ∼7-fold reduction in recurrence risk	GLP-1 RAs may accelerate ctDNA clearance; ctDNA kinetics offer a measurable end point for future GLP-1 RA + TNT trials
Operative outcomes	Weight loss (including semaglutide-induced) reduces visceral fat, shortens operative time, lowers bleeding, decreases conversion to open, reduces morbidity rate	GLP-1 RAs may optimize patients for surgery during TNT, improving perioperative and oncologic outcomes
Therapeutic timeline	TNT duration ∼6 months. GLP-1 RAs improve HbA1c in 3–6 months; weight loss plateaus at 9–15 months	Kinetics of GLP-1 RA effects align with TNT treatment window, supporting integration into protocols
Future directions	No clinical trials yet in rectal cancer. Hypothesis strongest in obese and/or T2D patients; potential role for GLP-1 receptor tumour expression profiling	Supports phase II RCTs with end points such as pCR, ctDNA clearance, disease-free survival, and organ preservation

TNT, total neoadjuvant therapy; HbA1c, glycated haemoglobin; GLP-1 RA, glucagon-like peptide-1 receptor agonist; DPP-4, dipeptidyl peptidase-4; cAMP, cyclic adenosine monophosphate; AKT, protein kinase B; ERK, extracellular signal-regulated kinase; CD8+ T cells, cluster of differentiation 8 positive cytotoxic T lymphocytes; Tregs, regulatory T cells; *ITPR1*, inositol 14,5-trisphosphate receptor type 1; pCR, pathological complete response; T2D, type 2 diabetes; ctDNA, circulating tumour DNA.

### Timing of GLP-1 RA use

Duration of TNT can vary, but as per the American Society of Colon and Rectal Surgeons it should not exceed 6 months^49^. RAPIDO uses a preoperative treatment phase of 6 months (4.5 months of active oxaliplatin-based chemotherapy). PRODIGE 23 uses 12 weeks of modified FOLFIRINOX then radiotherapy prior to surgery, again lasting about 6 months^50^. In clinical trials, and also in real-world data looking at GLP-1 RAs, the majority of improvement in HbA1c occurs in the first 3–6 months of therapy, with diminishing returns with continued dosing. Peak weight loss can take slightly longer, with time to weight loss plateau averaging more like 9–15 months with the more potent agents, although quicker for the less potent GLP-1 RAs^51–53^. We would postulate that the discussed molecular benefits of stimulating the GLP-1 receptors in the TME, such as a modulation of NF-κB and improved chemosensitivity, are much more rapid. The timeline to efficacy of GLP-1 RAs in their various beneficial effects fits alongside the duration of a standard TNT regime.

## Safety considerations in rectal cancer patients undergoing TNT

Although GLP-1 receptor agonists are generally well tolerated in metabolic populations, their safety profile warrants particular scrutiny in patients with rectal cancer undergoing TNT. Gastrointestinal adverse effects, including nausea, vomiting, diarrhoea, and delayed gastric emptying are among the most commonly reported toxicities in randomized trials^17,54,55^. In oncology patients, these effects may exacerbate treatment-related malnutrition, dehydration, and electrolyte imbalance, all of which are independently associated with chemotherapy interruption, dose reduction, and poorer outcomes^56–58^. GLP-1 RA-induced gastroparesis and delayed gastric emptying have prompted updated Anaesthesia Society guidance due to concern regarding increased aspiration risk^59^. In the perioperative setting, altered gut motility may theoretically compound the risk of postoperative ileus, particularly following pelvic surgery, although robust surgical outcome data in cancer populations remain limited^60^. Additionally, rapid weight loss and GLP-1 RA therapy have been associated with increased rates of gallbladder disease, including cholelithiasis and cholecystitis, and rare cases of pancreatitis have been reported^61,62^. Given that patients undergoing TNT frequently experience baseline sarcopenia, systemic inflammation, and treatment-related catabolism, the additive impact of these adverse effects warrants careful multidisciplinary oversight, structured nutritional monitoring, and clearly defined perioperative management protocols.

Current Anaesthesia Society guidance recommends withholding weekly GLP-1 receptors for at least 7 days prior to major surgery to mitigate aspiration risk related to delayed gastric emptying. In the context of TNT, this necessitates planned interruption prior to total mesorectal excision, with resumption considered postoperatively once oral intake and gastrointestinal function have stabilized. These perioperative considerations should be prospectively incorporated into clinical trial protocols evaluating GLP-1 RA use in rectal cancer^59^.

### Operative outcomes

Significant preoperative weight loss achieved through GLP-1 receptors may lead to measurable improvements in surgical logistics and patient outcomes. Evidence from bariatric and colorectal surgery indicates that reduced visceral adiposity facilitates clearer operative fields, simplifies fatty tissue handling, shortens procedure times, and decreases intraoperative bleeding rates^63–66^. Moreover, patients with lower BMI consistently experience lower conversion rates in minimally invasive rectal cancer surgery^67^.

High BMI is a well-known risk factor for postoperative complications such as wound infections, cardiopulmonary events, and delayed recovery^68^. In bariatric-preconditioned colorectal cancer patients, previous weight loss strategies have been associated with a 6.5% absolute reduction in postoperative morbidity rate as well as lower healthcare costs^69^. Although prospective data on semaglutide-specific effects are lacking, its ability to quickly reduce BMI provides a non-invasive way to lessen surgical risks and may improve rates of optimal surgical outcomes.

Although GLP-1 receptors agonists are well established for their role in weight reduction, their effects on glucose homeostasis and insulin sensitivity are also highly relevant in the perioperative setting. Reductions in perioperative hyperglycaemia significantly reduces thromboembolic events, helps wound healing, and reduces length of stay with fewer readmissions. Furthermore, the anti-inflammatory and cardioprotective effects of GLP-1 RAs could attenuate the systemic stress response of surgery, reducing the incidence of cardiopulmonary complications^70^. Optimizing metabolic and nutritional status with GLP-1 receptor agonists may therefore complement enhanced recovery after surgery (ERAS) pathways, facilitating earlier mobilization, reduced postoperative fatigue, and superior wound healing.

Although GLP-1 receptor agonists preferentially reduce adipose tissue, multiple body composition analyses have demonstrated that a proportion of total weight loss is attributable to reductions in lean mass. In the STEP trials and related semaglutide body composition substudies, approximately 25–40% of total weight loss was derived from lean tissue^17,54,71^. In oncology populations, even modest reductions in skeletal muscle mass are independently associated with increased chemotherapy toxicity, reduced treatment tolerance, postoperative morbidity rate, and inferior survival outcomes^72–74^. In rectal cancer patients undergoing TNT, baseline or treatment-associated sarcopenia has been linked to poorer oncologic and perioperative outcomes^75^. Given that TNT itself induces metabolic stress and catabolism, additional lean mass loss during GLP-1 RA therapy may exacerbate frailty and compromise oncologic resilience. Accordingly, if GLP-1 RAs are incorporated into this setting, structured nutritional assessment, resistance-based prehabilitation, and prospective body composition monitoring would be essential^74,76^.

## Future directions: planned phase II clinical trial

Building on this emerging rationale, our centre is preparing a multicentre, phase II, open-label RCT (registered on ClinicalTrials.gov (ClinicalTrials.gov Identifier: NCT07314528)) to formally evaluate the integration of GLP-1 RAs into TNT protocols for stage III rectal cancer. Eligible patients will be randomized to receive standard TNT alone or TNT combined with weekly semaglutide, commenced 4 weeks prior to FOLFOX and continued until surgery. The primary end point will be percentage weight change from baseline to post-TNT, with secondary outcomes including pathological complete response, disease-free and overall survival, local recurrence, circulating tumour DNA (ctDNA), and treatment-related morbidity and mortality. Adverse event monitoring will incorporate predefined surveillance for overlapping gastrointestinal toxicity, nutritional compromise, electrolyte disturbance, and treatment interruption.

**Fig. 1:**
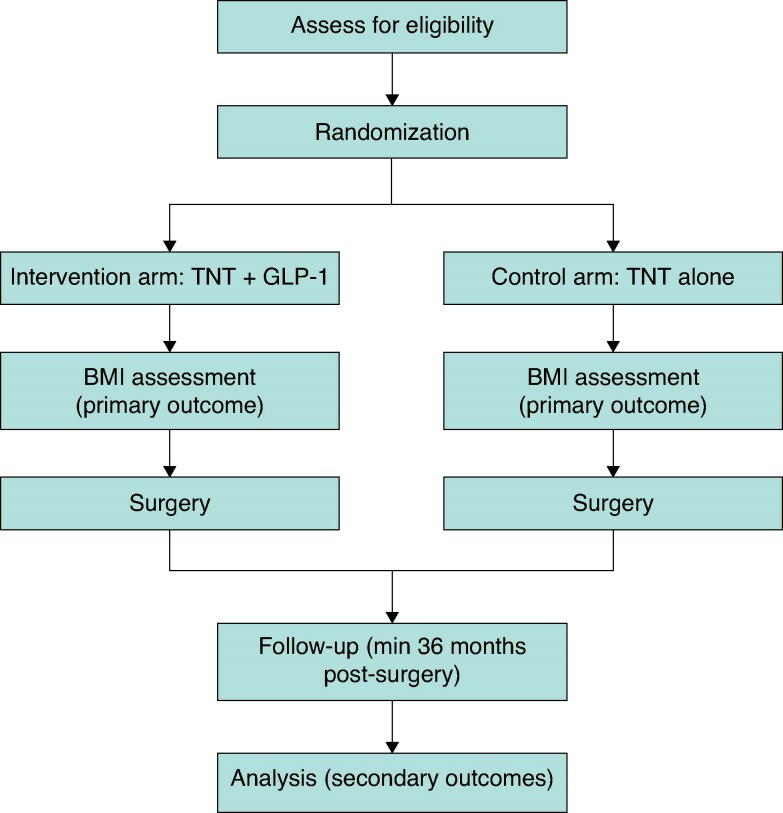
Study design of the proposed RCT comparing TNT plus GLP-1 RA *versus* TNT alone *Secondary outcomes: pathological complete response (pCR), complete clinical response (cCR), disease-free survival (DFS), pathological complete response (pCR), overall survival (OS), organ preservation, morbidity rate, and perioperative outcomes. Circulating tumour DNA (ctDNA) levels. *Figure reproduced under the terms of the Creative Commons Attribution 4.0 International License (CC BY 4.0), which permits unrestricted use, distribution, and reproduction in any medium, provided the original authors and source are credited.

This trial is designed to test whether metabolic modulation with semaglutide can enhance both treatment response and perioperative outcomes, and could provide the first prospective evidence to support GLP-1 receptor use as a dual-modality adjunct in rectal cancer management. To illustrate the trial design, we have developed a flowchart outlining patient randomisation, treatment arms, outcome assessments, and follow-up for the proposed study (*Fig. 1*).

## Conclusions

**Fig. 2:**
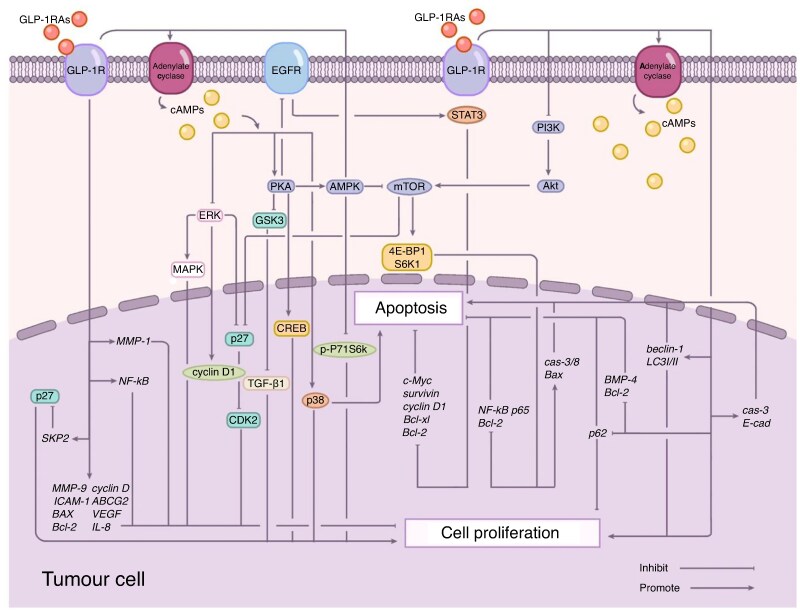
Molecular mechanisms of GLP-1 receptor agonist (GLP-1 RA)-mediated cancer cell growth inhibition^78^ GLP-1 RA binding to GLP-1 receptor triggers multiple signalling cascades that suppress cancer cell proliferation and promote apoptosis. The primary pathways include: (1) cAMP-mediated inhibition of ERK signalling, reducing cyclin D1 expression and DNA replication; (2) PKA-AMPK axis activation, leading to mTOR inhibition and p27-mediated cell cycle arrest; (3) cAMP-dependent activation of p38 pathway promoting apoptosis; (4) PKA-mediated suppression of EGFR-STAT3 signalling, downregulating prosurvival genes; (5) PI3K/Akt/mTOR pathway inhibition, increasing TGF-β1 levels and promoting cell cycle arrest; (6) NF-κB pathway suppression, reducing expression of proliferation-related genes; and (7) activation of autophagy pathways through regulation of beclin-1, LC3I/II, and p62 expression. Additional mechanisms include SKP2 inhibition, PGR upregulation, and BMP4 downregulation.

In conclusion, GLP-1 RAs are primarily established as antidiabetic and anti-obesity agents, with emerging preclinical and observational data suggesting potential effects on tumour biology, such as modulation of cell proliferation and survival. Still, these findings have not translated into clinical protocols for rectal cancer management^77^. Although GLP-1 RAs may have theoretical antineoplastic properties (*Fig. 2*), their integration into rectal cancer treatment remains investigational and unsupported by clinical data. A well-structured phase II clinical trial including pCR, ctDNA end points, and mechanistic biomarkers could measure their benefits and help guide larger studies focused on organ preservation strategies. Simultaneously, thorough safety evaluations and translational research will ensure that metabolic and oncologic advantages are achieved through a precision-based approach to rectal cancer treatment.

## Data Availability

All data generated or analysed during this study are included in the published article and its supplementary materials.

## Figures and Tables

All figures and tables are original to this manuscript and have not been published previously, except *Fig. 2*, which is reproduced under the terms of the *Creative Commons Attribution 4.0 International License (CC BY 4.0)*. This licence permits unrestricted use, distribution, and reproduction in any medium, provided the original authors and source are properly credited. *Figure 2* was reproduced from Lin, A., Ding, Y., Li, Z. *et al*. Glucagon-like peptide 1 receptor agonists and cancer risk: advancing precision medicine through mechanistic understanding and clinical evidence. *Biomarker Research*, 13, 50 (2025). Appropriate attribution and licensing conditions have been followed
